# Evolving techniques to optimize ERCP-based sampling and evaluation of malignant biliary strictures

**DOI:** 10.1093/gastro/goag067

**Published:** 2026-06-30

**Authors:** Chinmay Guralwar, Manik Aggarwal, Jyotroop Kaur, Vinay Chandrasekhara

**Affiliations:** Division of Gastroenterology and Hepatology, Department of Medicine, Mayo Clinic, Rochester, MN 55905, United States; Division of Gastroenterology and Hepatology, Department of Medicine, Mayo Clinic, Rochester, MN 55905, United States; Division of Gastroenterology and Hepatology, Department of Medicine, Mayo Clinic, Rochester, MN 55905, United States; Department of Internal Medicine, University of South Dakota Sanford School of Medicine, Sioux falls, SD 57108, United States; Division of Gastroenterology and Hepatology, Department of Medicine, Mayo Clinic, Rochester, MN 55905, United States

**Keywords:** cholangiocarcinoma, pancreatic ductal carcinoma, next-generation sequencing, DNA methylation, liquid biopsy, jaundice

## Abstract

Endoscopic retrograde cholangiopancreatography (ERCP)-based tissue acquisition is the cornerstone for assessment of suspected malignant biliary strictures. Conventional techniques, including biliary brush cytology and transpapillary forceps biopsy, are highly specific but are limited by modest sensitivity for detection of malignancy, often leaving a substantial proportion of individuals without a definitive diagnosis. To improve diagnostic sensitivity for cancer, several adjunctive approaches have been integrated into ERCP-based sampling. Peroral cholangioscopy (POC)-guided biopsies have become a reliable ERCP-based adjunctive modality, allowing direct visualization and targeted sampling, particularly in individuals with prior nondiagnostic procedures. Fluorescence *in situ* hybridization (FISH) is a technique used to improve diagnostic yield with biliary brushings by detecting chromosomal abnormalities, but is limited to specialized centers due to the need for specialized processing and training. Next-generation sequencing provides high sensitivity and can enable identification of actionable genomic alterations, while methylated DNA markers represent a promising, scalable molecular approach with strong diagnostic performance. Additional techniques include artificial intelligence–augmented direct visualization by POC and bile-based liquid biopsy. Current guidelines recommend multimodal ERCP-based sampling, combining at least two modalities where feasible, to optimize diagnostic accuracy. This review highlights the clinical performance, strengths, and limitations of established and emerging ERCP-guided techniques, emphasizing their evolving role in improving the diagnosis and management of malignant biliary strictures.

## Introduction

Extrahepatic malignant biliary obstruction (MBO) most often results from pancreatic cancer, cholangiocarcinoma (CCA), gallbladder cancer, and ampullary malignancy. Anatomically, extrahepatic MBO may be classified as perihilar and distal biliary strictures [1]. Early diagnosis of extrahepatic biliary malignancy is critical for maximizing the chance for curative management, but most malignant biliary strictures are diagnosed at an incurable stage. The core endoscopic methods for detecting hepatobiliary malignancies remain endoscopic retrograde cholangiopancreatography (ERCP) and endoscopic ultrasound (EUS) and both modalities are recommended for evaluation in all individuals with suspected extrahepatic MBO.

EUS provides high-resolution imaging of extrahepatic bile duct with added advantage of image guided sampling [2–4]. EUS-guided tissue sampling with fine needle aspiration or biopsy has demonstrated high sensitivity (90% for pancreatic ductal adenocarcinoma and 70%–80% for CCA) for distal malignant strictures. This is in part due to the anatomical position of the distal common bile duct immediately adjacent to the duodenum for easy visualization [2–4]. EUS guided sampling of perihilar masses can be challenging due to the anatomic distance of the hilum from the EUS transducer position in the duodenal bulb or stomach resulting in suboptimal imaging resolution and more difficult needle trajectory particularly due to intervening vessels. Perihilar CCA frequently grows in a periductal-infiltrating pattern with extensive desmoplastic reaction, making it difficult to identify a discrete targetable mass on EUS imaging, unlike the well-defined masses typically seen with distal strictures or pancreatic head tumors. In those perihilar strictures without a mass, direct sampling of the stricture is usually not pursued, but EUS can be valuable for assessing lymph nodes.

Most importantly, in those individuals that are potential resection or transplant candidates, EUS guided sampling of primary mass should not be performed as there is a risk of needle tract seeding which could preclude curative intent surgery. Most transplantation protocols consider prior transperitoneal sampling an absolute exclusion criterion, regardless of the biopsy result [5]. Therefore, ERCP-based intraductal sampling is necessary, particularly in those with perihilar strictures who may be candidates for curative resection or liver transplantation as EUS-guided sampling is contraindicated. Over the last two decades, molecular techniques, such as fluorescence *in situ* hybridization (FISH), next-generation sequencing (NGS), and methylated DNA markers (MDMs), have been developed to augment the diagnostic yield of samples obtained with biliary brushings and transpapillary forceps biopsy (TPB). Advancements in direct visualization and targeted sampling with peroral cholangioscopy (POC) have further enhanced diagnostic accuracy. More recently, artificial intelligence (AI)-enhanced POC and multiomic profiling of bile represent additional tools that can enhance diagnostic sensitivity with ERCP. This review aims to summarize the established and emerging ERCP-based diagnostic modalities by highlighting their accuracy and potential clinical role in the evaluation of malignant biliary strictures.

## Current standard of care

### Brush cytology

**Figure 1:**
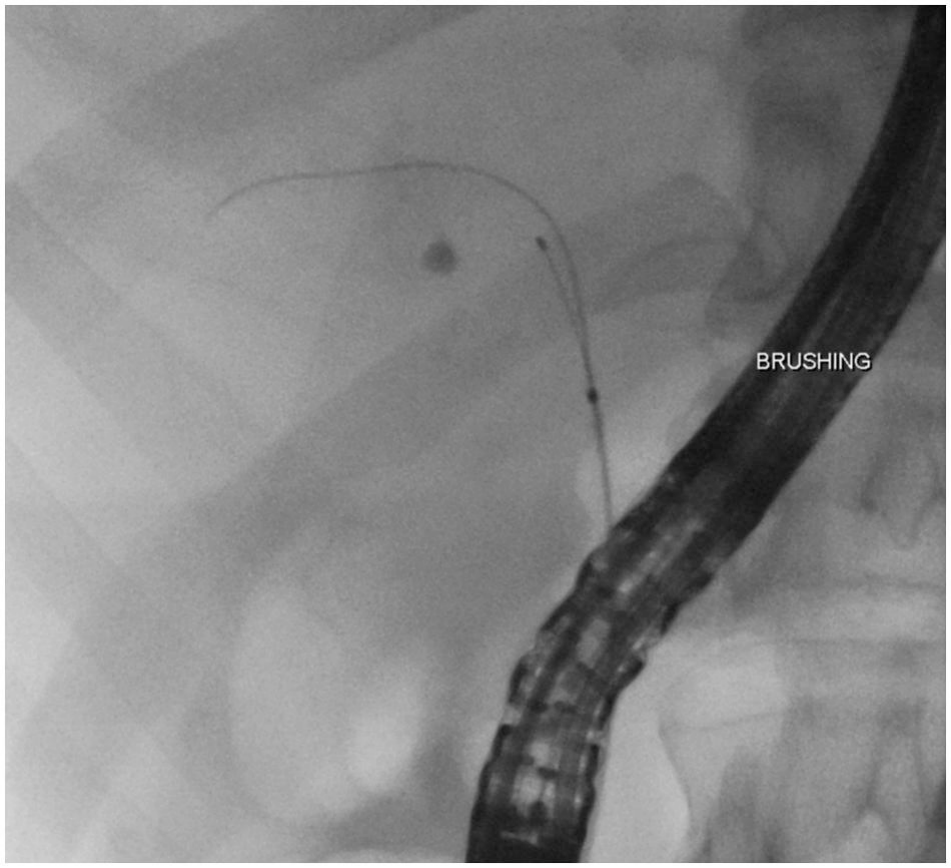
Fluoroscopic image of intraductal brushing by wire guided biliary brush.

Brush cytology is widely available, relatively easy to perform, and is routinely used as a first diagnostic step. It involves passing a wire-guided cytology brush within a double lumen catheter sheath into the bile tree, advancing the brush from the catheter, and then moving it back and forth across the stricture to collect epithelial cells for cytological evaluation (Fig. 1). The results are typically categorized as negative for malignancy, atypical, suspicious for malignancy, or positive for malignancy [6, 7].

ERCP with brush cytology is highly specific but limited by poor sensitivity (19%–60%) for the detection of biliary malignancy [8–11]. This is primarily a result of the desmoplastic nature, longitudinal growth pattern of CCA, and technical difficulties in obtaining targeted samples [12]. Cellular yield can be improved by using a minimum set threshold of brush passes through the entire stricture, removing the catheter and brush together to avoid brush withdrawal through the length of the catheter, and flushing residual cells from within the catheter into the sample vial after removal of the brush. In a randomized comparative study, 30 brush passes were associated with a higher diagnostic sensitivity without an increased risk of adverse events (AEs) when compared to 10 brush passes, suggesting a higher number of passes may improve diagnostic yield of brush cytology [13]. Over the last two decades, strategies to improve performance, such as stricture dilation prior to brushing, novel brush design, and rapid onsite evaluation (ROSE) for biliary brushing specimens, have been proposed but none so far have been widely adopted [14–16].

Despite ease of use and wide availability, brush cytology is often inadequate to solely guide clinical decision making due to low diagnostic sensitivity. Therefore, additional diagnostic modalities should be employed during index ERCP, when feasible. In addition, use of newer AI-based tools for cytology interpretation may significantly reduce interpretation time while maintaining similar diagnostic performance as shown in a recent study [17]. Future validation in larger cohorts and in varied slide preparations will assist in understanding the utility of AI in cytology.

### Fluoroscopy-guided transpapillary forceps biopsy

**Figure 2:**
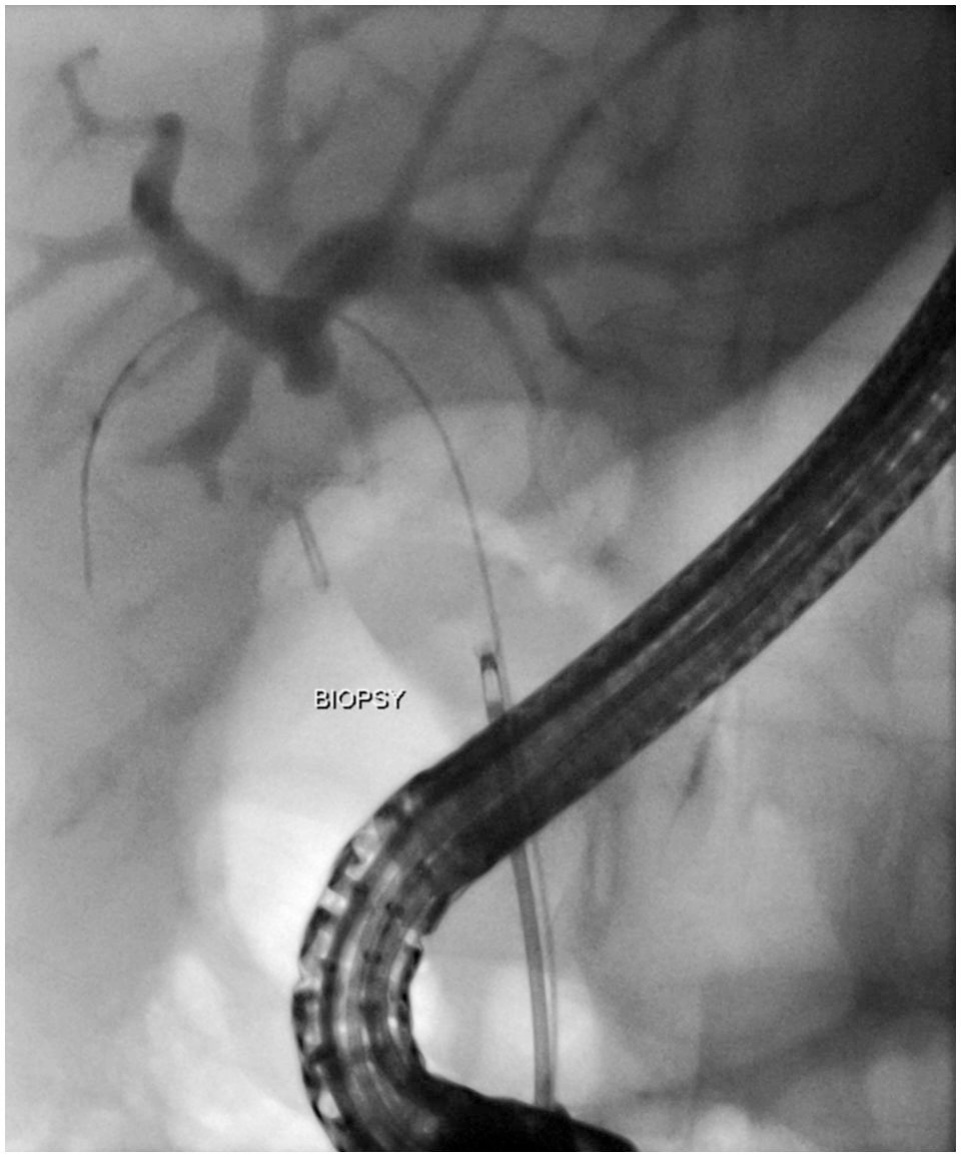
Fluoroscopic image of transpapillary forceps biopsy being performed for evaluation of common hepatic duct biliary stricture.

ERCP-directed TPB has modest sensitivity (40%–88%) for the detection of biliary malignancy [18–21]. This technique involves tissue sampling by advancing biopsy forceps under fluoroscopy guidance through the accessory channel of a duodenoscope to the site of the stricture (Fig. 2). The forceps are then used to obtain multiple tissue samples directly from the stricture for histopathological evaluation. Although there is also some uncertainty regarding the optimal number of biopsies, higher diagnostic sensitivity is seen with increasing the number of tissue samples with a threshold of at least three to five samples [22–24]. In our practice, we typically use standard pediatric cold forceps (Radial Jaw 4 Pediatric Biopsy Forceps, Boston Scientific Corporation, Marlborough, Massachusetts, United States) which is advanced into the bile duct under fluoroscopic guidance to the site of the stricture. We typically obtain three or more biopsy specimens with 1–2 “bites” per pass. The tissue specimen is fixed with formalin and submitted for pathological review. The clinical utility of fluoroscopic-guided TPB lies in its ability to provide histological diagnosis, which is superior to brush cytology alone for architectural assessment, and combination of these two modalities has shown a significant improvement in sensitivity for diagnosis of malignant strictures as compared to brush cytology alone [25–29].

Developments, such as using large capacity forceps compared to the standard forceps (2.2 vs 1.8 mm) and using 90-degree adjustable biopsy forceps, have also shown to increase sensitivity to 70% vs 43% and 71.4% vs 66.7%, respectively [18, 19]. Some evidence suggests improved sensitivity of 71% as compared to 41% when using balloon dilation prior to TPB [30].

Technical difficulty in accessing tight or angulated strictures and the need for advanced endoscopic skill may limit the generalizability of most studies outside of tertiary care centers. Furthermore, TPB relies on two-dimensional fluoroscopic imaging, and the directionality of the forceps makes it challenging to know if the area of concern is being sampled. Nevertheless, the routine use of fluoroscopy guided TPB in conjunction with brush cytology at index ERCP evaluation improves diagnostic yield without a significant increase in AEs and is recommended in the guidelines by the American Society for Gastrointestinal Endoscopy (ASGE), European Society for Gastrointestinal Endoscopy (ESGE), and Asia-Pacific consensus group [31–33].

### Fluorescence *in situ* hybridization

FISH is a molecular cytogenetic technique utilizing fluorescent labeled DNA probes to detect specific structural chromosomal abnormalities in cells obtained from biliary brushings. FISH is highly sensitive for identifying submicroscopic alterations that are not visible by conventional karyotyping, such as microdeletion syndromes (e.g. 22q11 deletion), and is routinely used to detect specific gene fusions, copy number changes, and chromosomal rearrangements in both constitutional and neoplastic disorders.

**Figure 3:**
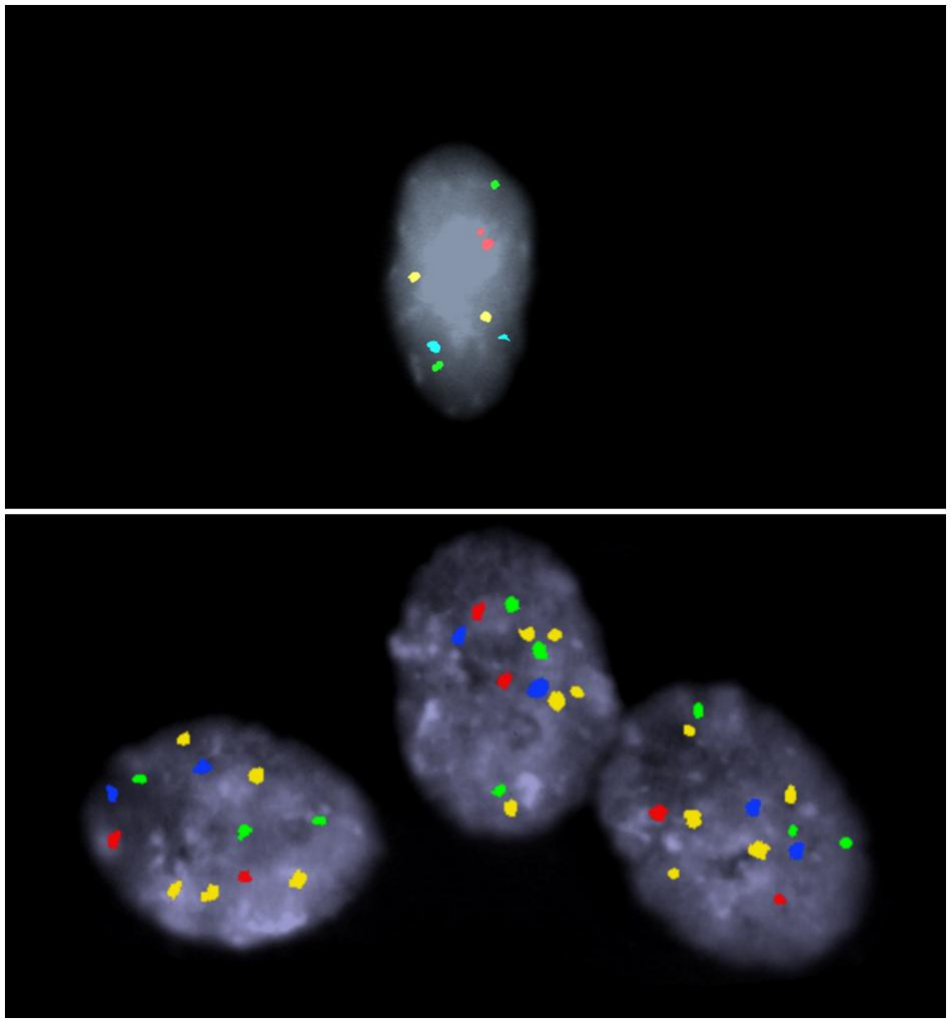
Pancreatobiliary fluorescence *in situ* hybridization (FISH) probe set targeting 1q21, 7p12, 8q24, and 9p21: benign cell demonstrating disomy (two copies per locus) (top); malignant cells demonstrating polysomy (copy number gain at multiple loci) (bottom).

FISH has emerged as a useful adjunct to brush cytology since the initial report of using the Urovysion probe set (Abott Molecular Inc., Des Plaines, IL, USA), originally developed for urothelial malignancy, on biliary brushing specimens. Polysomy (defined as gain of multiple chromosomes) on the Urovysion probe set demonstrated a sensitivity of 42.9% compared to 20.1% for brush cytology in 498 ERCP brushing samples, with comparable specificity [34]. Further development of an optimized set of FISH probes for pancreatobiliary malignancies, targeting 1q21, 7p12, 8q24, and 9p21 loci, demonstrated a significantly higher sensitivity of 65% than the Urovysion probe set. The Pancreatobiliary FISH probe set has subsequently been used in our clinical practice for evaluation of biliary strictures (Fig. 3). Our practice is to obtain one biliary brushing specimen from the stricture (multiple brushings in case of multiple strictures) and split the obtained specimen for cytology and FISH evaluation [35]. The utility of FISH is particularly evident in individuals with primary sclerosing cholangitis (PSC), where serial or multifocal polysomy has been shown to strongly correlate with CCA [36–38]. The addition of FISH to brush cytology showed improvement in sensitivity (44%–69%) compared to cytology alone (17%–54%) [39–42]. However, certain abnormalities, such as trisomy or tetrasomy in the absence of polysomy, may yield false positive results, especially in inflammatory conditions like PSC, emphasizing the need for careful interpretation [43]. FISH polysomy in isolation without other suggestive clinicoradiological features should not be used to guide oncologic therapy. Additionally, the technique requires specialized cytopathological expertise for both performance and interpretation, limiting its widespread clinical adoption. Based on the increased sensitivity, all major guidelines recommend the use of FISH as an adjunct to conventional tissue sampling, especially when initial testing is non-diagnostic [1, 31, 38].

## Intraductal imaging

### Single operator peroral cholangioscopy

POC enables direct visualization of the biliary tree and targeted intraductal biopsies, overcoming the limitations of fluoroscopy guided sampling. Single-operator POC, performed using an ultra-slim flexible endoscope, has largely supplanted the earlier two-operator mother-daughter cholangioscopy system. POC, in an adjunctive role, can lead to improved diagnostic accuracy, better define the extent of disease, and aid in surgical planning.

#### Direct visualization and peroral cholangioscopy-guided sampling

**Figure 4:**
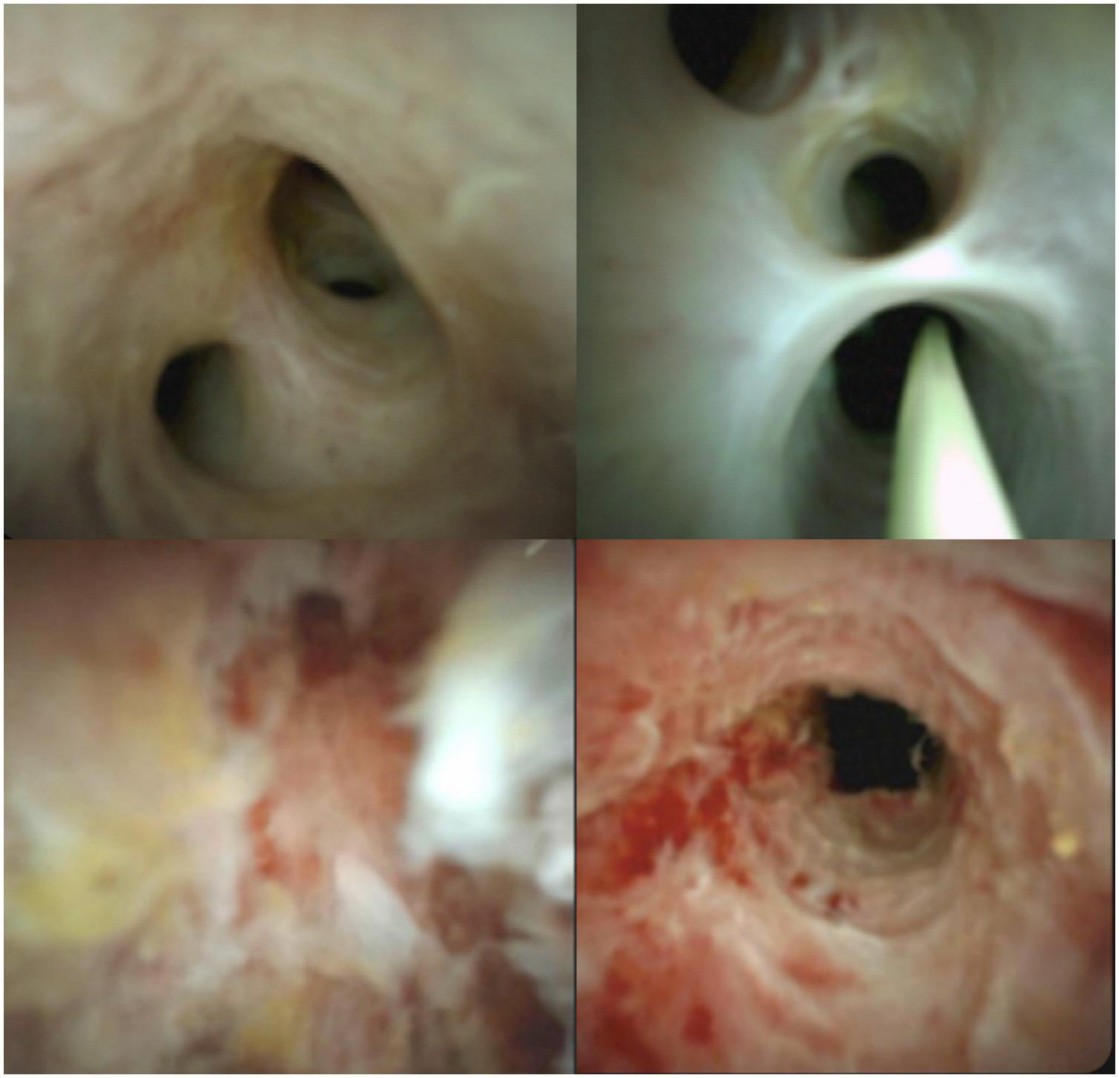
Single operator peroral cholangioscopy showing benign ducts (top left and right). Single operator peroral cholangioscopy showing abnormal vasculature, ulcerations, and exudative mucosa (bottom left and right).

Strictures visualized with POC should be carefully inspected for features suggestive of malignancy (Fig. 4). Several classification systems have been developed in an attempt to better standardize detection of malignancy. The Monaco classification was developed using a consensus process involving expert interventional endoscopists (Table 1) [44]. It consists of eight visual criteria for characterizing biliary lesions which include the presence of a stricture, lesion, mucosal features (granular or irregular), papillary projections (uniform or fingerlike), ulceration, abnormal vessels (tortuosity), scarring (white linear bands or rings) and pronounced pit pattern. Although the Monaco classification demonstrated an overall diagnostic accuracy of 70% for distinguishing malignant from benign strictures, it was limited by high interobserver variability for each individual criterion. The Monaco classification was further refined into five criteria labeled as the Mendoza classification (Table 1) [45]. In the Mendoza classification, there was improved interobserver agreement for features associated with malignancy, including tortuous and dilated vessels, raised intraductal lesions, and friability. Other adjunctive technologies, such as narrow-band imaging, may enhance mucosal and microvascular visualization, potentially improving diagnostic accuracy for malignant biliary strictures [46, 47]. However, none of these classifications have been uniformly accepted and require validation in larger studies before widespread adoption. While the visual cholangioscopic impression demonstrates moderate sensitivity, the specificity may be suboptimal in the absence of tissue acquisition which remains the standard for oncologic decision-making [48–50]. Moreover, cholangioscopy can be technically challenging in distal biliary strictures as the system is often unstable in the periampullary location and tends to migrate out of the duct making visualization more difficult [51].

**Table 1:** Visual criteria of the Monaco and Mendoza classifications for digital single-operator cholangioscopy

	Monaco classification		Mendoza classification
1	Presence of stricture, and if stricture was asymmetric or symmetric	1	Presence of tortuous and dilated vessels
2	Presence of lesion, and if lesion had a mass greater than one-fourth diameter of the duct, or a nodule (size less than one-fourth diameter of the duct), or had a polypoid appearance.	2	Presence of irregular nodulations
3	Mucosal features that were either smooth or granular.	3	Presence of raised intraductal lesion
4	Papillary projections, and whether these projections were fingerlike (long) or short.	4	Presence of irregular surface with or without ulcerations
5	Ulceration	5	Presence of friability
6	Abnormal vessels		
7	Scarring, and whether scarring was local or diffuse.		
8	Pronounced pit pattern		

**Figure 5:**
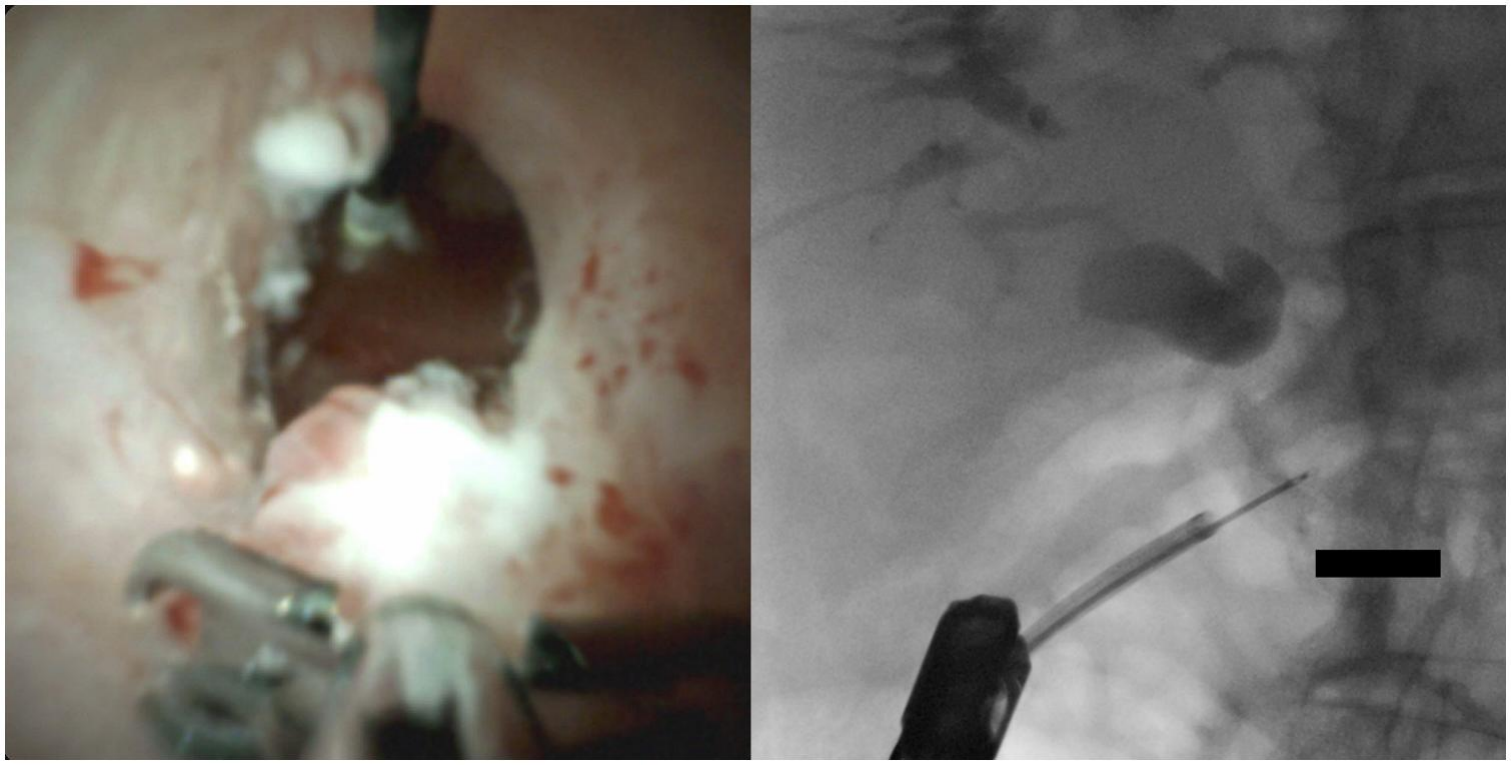
Cholangioscopy guided targeted intraductal biopsy of biliary stricture (left). Fluroscopic image of cholangioscopy guided targeted intraductal biopsies (right).

Beyond visual assessment, cholangioscopy allow for targeted biopsies of an area of concern (Fig. 5). In a randomized controlled trial of 57 patients with indeterminate strictures, POC-guided biopsy achieved a sensitivity of 68.2% compared with 21.4% for ERCP-guided biopsy, both with 100% specificity [52]. A meta-analysis on efficacy of POC in the diagnosis of indeterminate biliary strictures involving 356 individuals reported a pooled sensitivity of 74% and specificity of 98%, with an AUC close to 0.95, indicating excellent overall accuracy [53]. In proximal strictures previously nondiagnostic by TPB, POC-guided biopsy achieved a sensitivity of 92.3% [54]. Tissue acquisition has historically been constrained by small working channels, but newer systems with larger channels (up to ∼1.7 mm) may permit larger accessories and improve sampling adequacy [55, 56].

When standard ERCP sampling fails, cholangioscopy provides meaningful additional diagnostic information with sensitivity of 60%–75% in previously sampled strictures. A recent ASGE guideline notes that cholangioscopy is “especially important with prior nondiagnostic ERCP without cholangioscopy” [33]. However, data suggest that upfront POC guided sampling may increase diagnostic yield over traditional brushings-based cytology alone. In a multicenter randomized controlled trial of 60 individuals, POC at index ERCP achieved first-sample sensitivity of 68.2% versus only 21.4% for standard ERCP brushing [52]. Additionally, prior manipulation of biliary strictures through stent placement or prior tissue acquisition may decrease the sensitivity and accuracy of subsequent cholangioscopy-guided biopsies [57]. It is important to note that adjunctive molecular techniques, such as FISH, which are recommended by guidelines [1], were not routinely performed in these studies and thus may underestimate the performance of standard brushings-based techniques without POC.

The promise of faster diagnosis, reduced likelihood of repeat procedures by using upfront POC is further tempered by limited availability of POC to high-volume centers primarily due to the need for specialized technical expertise. Adequate sampling with POC requires greater technical skill and likely longer procedure times with known complications associated with POC. Another major limitation of POC remains the high upfront procedural costs (∼$3,530 vs. $893 for standard ERCP based on ASGE guidelines) [1]. However, modelling studies have demonstrated that upfront POC may be cost effective at standard willingness-to-pay thresholds given the frequent need for repeat diagnostic procedures [58].

Our current practice is personalized to the clinical scenario. We proposed that the decision to perform index POC should be individualized based on: (i) stricture location (particularly in hilar strictures which are known to be particularly difficult to sample); (ii) availability of adjunctive brushing-based techniques, such as FISH or NGS; (iii) availability of POC expertise at the performing center; and (iv) clinical urgency for diagnosis. Table 2 summarizes studies from 2020 onwards evaluating performance of POC-guided testing modalities for the assessment of biliary strictures.

**Table 2:** Diagnostic performance of peroral cholangioscopy-guided testing modalities for malignant biliary strictures (2020 to present)

Study	Year	Study design	Country	Total cohort (*n*)	Individuals with malignancy (*n*)	Cholangioscopy platform	Testing modality	Sensitivity (%)	Specificity (%)
de Vries *et al.* [49]	2020	Retrospective	Netherlands	57	20	SpyGlass DS II	POC-guided biopsy	15.0	65.0
				80	22	SpyGlass DS II	POC-guided visualization	64.0	62.0
Pereira *et al.* [50]	2020	Retrospective	Portugal	43	24	SpyGlass DS	POC-guided biopsy	63.6	100
				43	24	SpyGlass DS	POC-guided visualization	100	89.5
Jang *et al.* [59]	2020	Retrospective	USA	101	N/A	SpyGlass DS	POC-guided biopsy	69.8	97.9
				105	55	SpyGlass DS	POC-guided visualization	89.1	90.0
Almadi *et al.* [60]	2020	Prospective	Multicenter^a^	163	N/A	SpyGlass Legacy, SpyGlass DS	POC-guided biopsy	75.3	100
				289	113	SpyGlass Legacy, SpyGlass DS	POC-guided visualization	86.7	71.2
Kaura *et al.* [36]	2020	Retrospective	USA	64	N/A	SpyGlass Legacy, SpyGlass DS	POC-guided biopsy	43.3	97.1
				92	N/A	SpyGlass Legacy, SpyGlass DS	POC-guided visualization	54.0	81.0
Robles-Medranda *et al.* [61]	2020	Retrospective	Ecuador	147	70	SpyGlass DS	POC-guided visualization	91.0	99.0
Onoyama *et al.* [62]	2020	Retrospective	Japan	31	18	SpyGlass DS	POC-guided biopsy	83.3	100
Gerges *et al.* [52]	2020	RCT	Multicenter	31	22	SpyGlass DS	POC-guided biopsy	68.2	100
Bang *et al.* [63]	2020	RCT	USA	30	13	SpyGlass DS	POC-guided biopsy (off-site group)	76.9	100
				32	16	SpyGlass DS	POC-guided biopsy (on-site group)^b^	75.0	100
Han *et al.* [25]	2021	Retrospective	USA	83	N/A	Video Narrow Band Imaging Choledochoscope and Choledochofiberscope, fiberoptic SpyGlass Direct Visualization System, Spyglass DS	POC-guided biopsy	51.1	97.4
				111	N/A	Video Narrow Band Imaging Choledochoscope and Choledochofiberscope, fiberoptic SpyGlass Direct Visualization System, Spyglass DS	POC-guided visualization	82.1	67.3
Weigand *et al.* [64]	2021	Retrospective	Germany	161	31	SpyGlass DS	POC-guided biopsy	87.0	88.0
Fugazza *et al.* [65]	2022	Prospective	Italy	201	N/A	SpyGlass DS	POC-guided biopsy	80.2	92.6
				201	N/A	SpyGlass DS	POC-guided visualization	88.5	77.3
Sekine et al. [66]	2022	Retrospective	Japan	59	50	SpyGlass Legacy, SpyGlass DS	POC-guided biopsy	54.0	100
Bokemeyer et al. [67]	2022	Retrospective	Germany	12	N/A	SpyGlass DS	POC-guided biopsy	50.0	100
				22	4	SpyGlass DS	POC-guided visualization	75.0	94.4
Büringer *et al.* [68]	2024	Retrospective	Germany	108	93	SpyGlass DS	POC-guided biopsy	86.0	99.0
Robles-Medranda *et al.* [69]	2024	Retrospective	Ecuador	32	N/A	eyeMAX	POC-guided biopsy	95.8	100
				49	N/A	eyeMAX	POC-guided visualization	91.6	87.5
Milluzzo *et al.* [70]	2024	Retrospective	Italy	14	10	SpyGlass DS	POC-guided biopsy	44.4	100
				14	10	SpyGlass DS	POC-guided visualization	88.9	80.0
Tejido *et al.* [71]	2024	Retrospective	Spain	28	N/A	SpyGlass Legacy, SpyGlass DS, SpyGlass DS II	POC-guided biopsy	36.4	100
				61	27	SpyGlass Legacy, SpyGlass DS, SpyGlass DS II	POC-guided visualization	100	91.2
de Jong *et al.* [57]	2025	Prospective	European Cholangioscopy Group^c^	80	50	SpyGlass DS II	POC-guided biopsy (single biospy group)	66.0	100
				76	47	SpyGlass DS II	POC-guided biopsy (bite-on bite biopsy group)	63.8	100
Ogura *et al.* [72]	2025	Prospective	Japan	50	23	eyeMAX	POC-guided biopsy	91.3	96.3
							POC-guided visualization	91.3	100
				47	27	SpyGlass DS II	POC-guided biopsy	85.2	95.0
							POC-guided visualization	66.7	100

POC = peroral cholangioscopy, N/A = not applicable or not reported in the original article, RCT= randomized controlled trial.

a Australia, Hong Kong, India, Japan, Pakistan, Saudi Arabia, Singapore, South Africa, South Korea, Thailand.

b Rapid on-site evaluation of specimens by the touch imprint cytology technique.

c Belgium, Bulgaria, Finland, Germany, Italy, Netherlands, Spain, United Kingdom.

### AI-enhanced cholangioscopy

**Figure 6:**
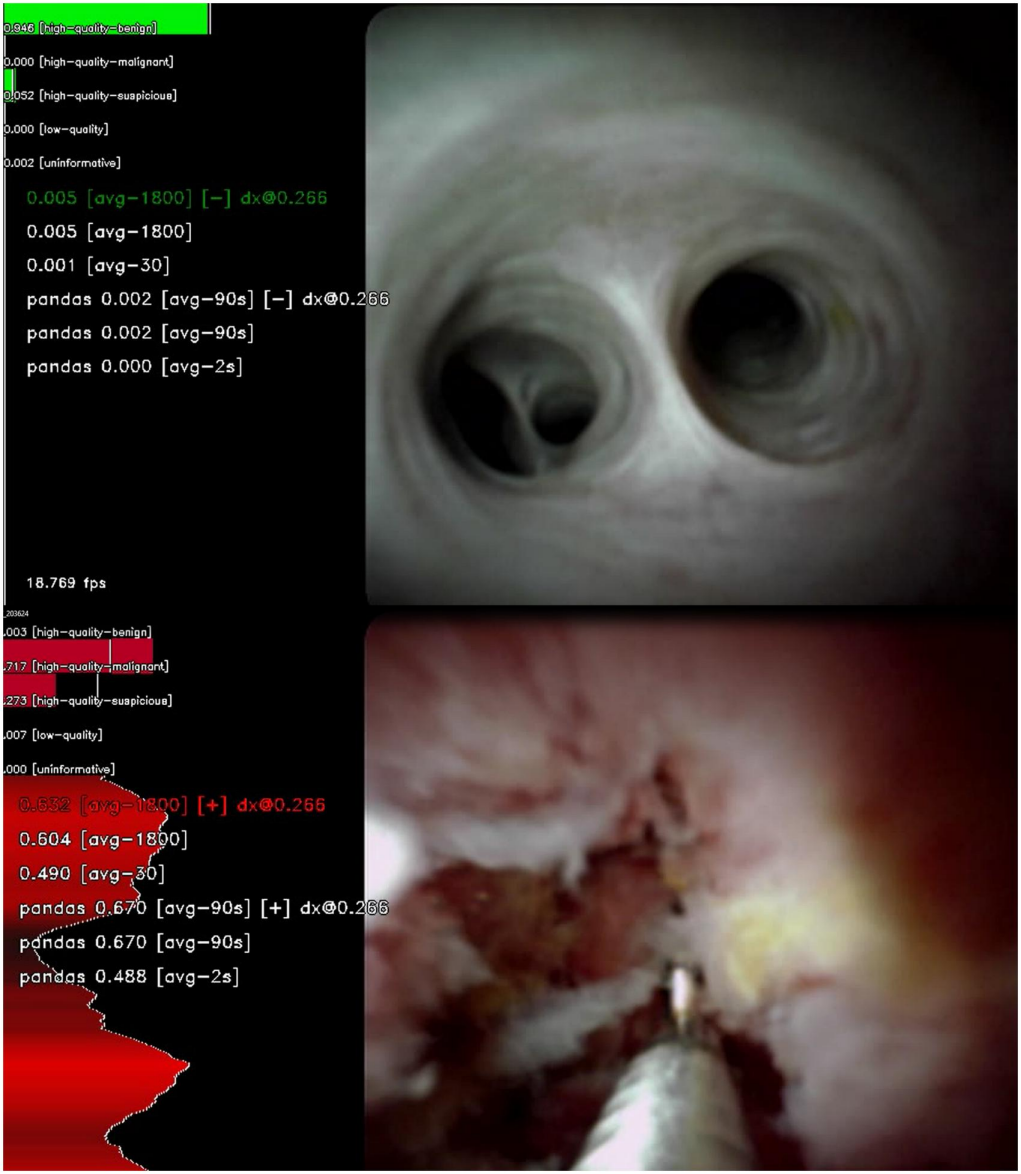
Real-time artificial intelligence assisted cholangioscopic evaluation of biliary strictures; benign stricture (top), malignant stricture (bottom).

The subjective nature of visual assessment and the poor interobserver agreement with cholangioscopy imaging interpretation have limited widespread adoption. AI offers a means to standardize interpretation and potentially enhance the diagnostic performance of POC (Fig. 6). Several groups have developed AI models to differentiate benign and malignant strictures on POC. In a study of 154 individuals undergoing POC, a convolutional neural network (CNN) model achieved an accuracy of 91% and an AUC of 0.94, significantly outperforming conventional ERCP-based brush cytology (62.5%) and forceps biopsy (60.9%) [73]. CNN-based models have been demonstrated to improve diagnostic accuracy for both cholangioscopy experts and non-experts [74]. AI-based models can also recognize specific morphological features associated with malignancy (nodules, papillary projections, and tumor vessels) and guide directed sampling [75].

Taken together, these studies indicate that AI-assisted cholangioscopy outperforms unaided visual interpretation in differentiating benign from malignant strictures. However, current models are largely based on retrospectively collected, still-image or limited video datasets, are trained against heterogeneous reference standards, and have not yet been fully deployed or evaluated in real-time clinical workflows. Future research priorities include prospective multicenter video-based validation, harmonization of annotation and outcome definitions, and rigorous assessment of how AI integration affects diagnostic yield, procedure time, training, and downstream clinical decision-making in everyday practice.

## Role of multi-modal assessment

Individual diagnostic modalities are limited by suboptimal sensitivity for detecting biliary tract malignancy. This may be partly mitigated by using complementary multimodality diagnostic approaches to maximize overall diagnostic yield [1, 38]. Over the past two decades, several studies have consistently demonstrated incremental improvement in diagnostic sensitivity with addition of diagnostic modalities to brush cytology, without compromising specificity. Stricture evaluation using brush cytology in combination with two supplementary approaches, namely, intraductal biopsy (guided by either fluoroscopy or POC) and FISH, appears to achieve optimal sensitivity.

This triple-modality approach significantly improves sensitivity from 68.3% to 82% for diagnosis of malignant biliary stricture [76, 77]. In a subsequent study, progressive increase in diagnostic sensitivity were noted with addition of FISH to cytology and POC to FISH and cytology [36]. Similar results were seen in a larger cohort of 204 individuals, where a significant increase in diagnostic sensitivity was seen with addition of each modality rising from 17.3% alone for brush cytology alone to 40.4% in combination with TPB, and 58.7% with FISH, and ultimately 68.3% when all three techniques were combined [42]. Combining cholangioscopy to brush cytology and FISH has consistently been shown to provide the highest diagnostic performance with sensitivity rising to 80.4% while maintaining high specificity in those indeterminate biliary strictures [25].

**Figure 7:**
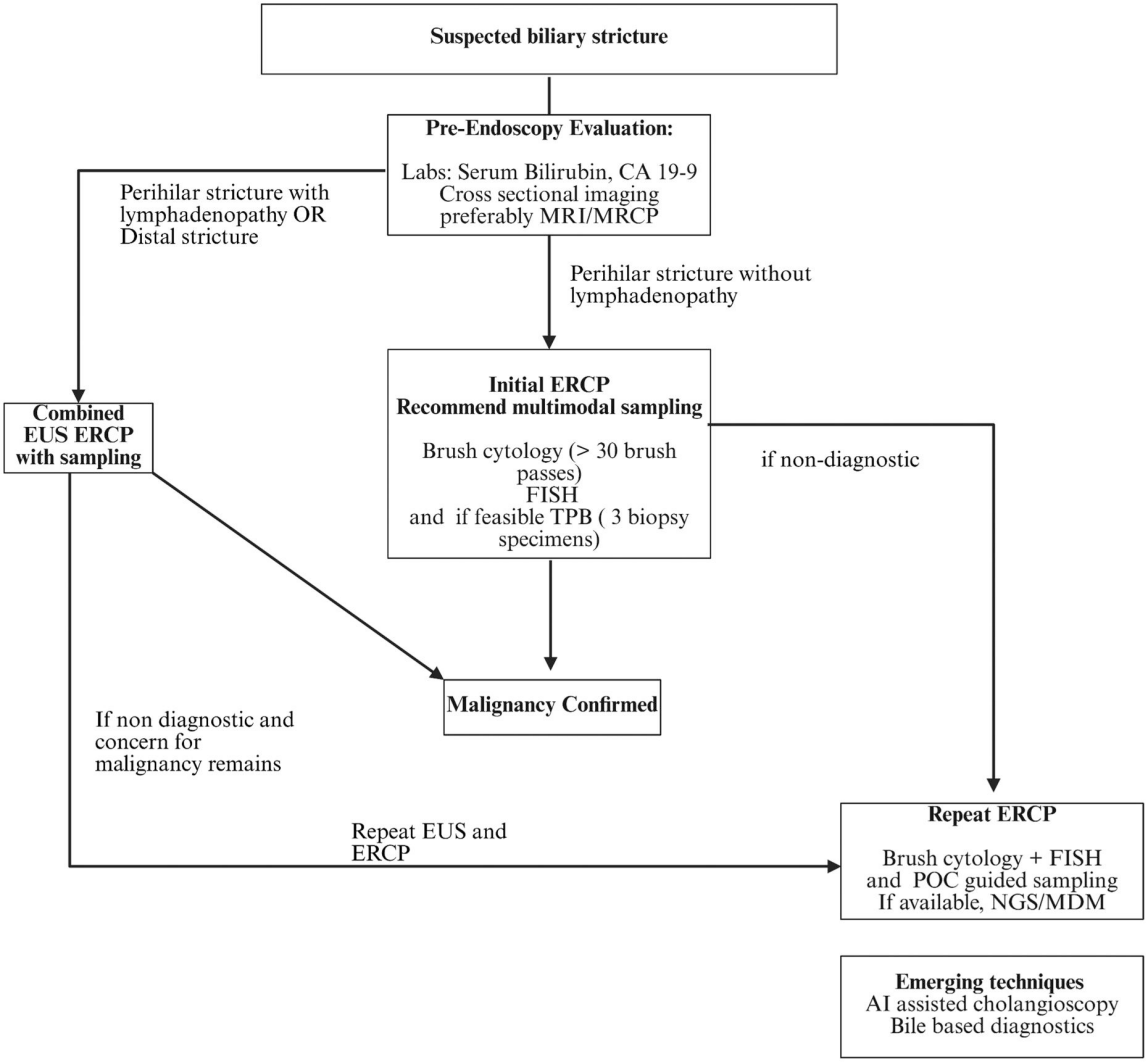
Diagnostic algorithm for indeterminate biliary strictures. MRI = magnetic resonance imaging, MRCP = magnetic resonance cholangiopancreatography, EUS = endoscopic ultrasound, ERCP = endoscopic retrograde cholangiopancreatography, PB-FISH = pancreatobiliary fluorescence *in situ* hybridization, TPB = transpapillary forceps biopsy, POC = peroral cholangioscopy, NGS = next-generation sequencing, MDM = methylated DNA markers.

Reflecting on this evidence, international guidelines, including those from the ASGE, ESGE, and Asia-Pacific consensus groups, recommend combining intraductal biopsies and brush cytology at index ERCP to confirm the etiology of malignant biliary stricture [31–33]. The ACG clinical guidelines recommend a multimodal approach for diagnosing malignant biliary strictures, using at least two sampling techniques and using three to four modalities at the initial evaluation if they can be performed safely [1]. Table 3 summarizes original studies published from 2015 onward evaluating multimodal diagnostic approaches for the assessment of biliary strictures. A suggested diagnostic algorithm for diagnosis of biliary strictures is shown in Fig. 7.

**Table 3:** Diagnostic performance of multimodal assessment strategies for malignant biliary strictures (2015 to present)

Study	Year	Study design	Country	Total cohort (*n*)	Individuals with malignancy (*n*)	Sample type	Testing modality	Sensitivity (%)	Specificity (%)
Nanda *et al.* [78]	2015	Retrospective	USA	50	22	Brushing/Biopsy	Combination of brush cytology, fluoroscopy guided TPB and FISH	82.0	100
Naitoh *et al.* [76]	2016	Retrospective	Japan	241	179	Brushing/Biopsy	Combination of brush cytology and fluoroscopy guided TPB	69.1	100
Chaiteerakij *et al.* [79]	2016	Prospective	Thailand, USA	99	93	Brushing	Combination of brush cytology and FISH	59.1	100
Gonda *et al.* [39]	2017	Prospective	USA	100	41	Brushing	Combination of brush cytology and FISH	51.0	100
Liew *et al.* [40]	2018	Retrospective	Singapore	30	20	Brushing	Combination of brush cytology and FISH	69.2	82.4
Kushnir *et al.* [41]	2019	Prospective	USA	101	70	Brushing	Combination of brush cytology and FISH	44.0	100
Kaura *et al.* [36]	2020	Retrospective	USA	92	41	Brushing/Biopsy	Combination of brush cytology, fluoroscopy guided TPB, POC-guided biopsy, and FISH	69.2	82.6
Chang *et al.* [77]	2020	Retrospective	China	41	35	Brushing/Biopsy	Combination of brush cytology and fluoroscopy guided TPB	77.1	100
Han *et al.* [25]	2021	Retrospective	USA	614	192	Brushing/Biopsy	Combination of brush cytology, POC-guided biopsy, and FISH	80.4	67.3
Baroud *et al.* [42]	2022	Retrospective	USA	204	104	Brushing/Biopsy	Combination of brush cytology, fluoroscopy guided TPB and FISH	68.3	92.0
Cooley *et al.* [80]	2024	Prospective	USA	197	64	Brushing	Combination of brush cytology and FISH	68.3	96.9

FISH = fluorescence in situ hybridization, POC = peroral cholangioscopy, TPB = transpapillary forceps biopsy.

## Molecular diagnostic techniques

Molecular assays applied to ERCP-obtained specimens address a central limitation of conventional cytology and biopsy: low sensitivity in desmoplastic, paucicellular, and longitudinally infiltrative biliary tract malignancies. These approaches can be performed on tissue (brushings/biopsies) or on bile as a liquid biopsy matrix and are best viewed as adjuncts that increase diagnostic confidence and, potentially, provide therapeutically actionable information.

### Next-generation sequencing

NGS applied to ERCP-derived brushings or TPB specimens increases diagnostic sensitivity by detecting cancer-associated genomic alterations. In a prospective study of 252 individuals, a 28-gene tissue NGS panel demonstrated 73% sensitivity with 100% specificity for diagnosing biliary malignancy [81]. In a single-center retrospective study of 77 individuals, addition of NGS panel to cytology/TPB resulted in an increased sensitivity to 85.7% versus 42.9% for cytology and TPB alone [82].

NGS may be particularly useful in PSC, where inflammatory atypia and multifocal disease affect the performance of conventional sampling; a 14-gene tissue panel reported 75% sensitivity in a PSC cohort enriched for malignancy [83]. Beyond diagnosis, tissue NGS can identify potentially actionable alterations (e.g. *TP53*, *BRAF*, *CTNNB1*, *SMAD4*, *KRAS/NRAS*), supporting downstream treatment stratification when tissue is otherwise limited; these alterations have been detected across cytology and bile-derived material, although variant allele frequencies may be lower in bile [84].

While promising, several factors must be considered when assessing NGS panels as variability exists due to panel composition, laboratory pipelines, sample adequacy, and cohort prevalence, which complicates cross-study comparisons (Table 4). With standardization of sample collection, assay development, and reporting, the use of NGS panels may increase in the future; however, lack of insurance reimbursement remains a major obstacle to more widespread adoption.

**Table 4:** Diagnostic performance of Next Generation Sequencing panels for malignant biliary strictures (2015 to present)

Study	Year	Study design	Country	Total cohort (n)	Individuals with malignancy (n)	Sample type	Testing modality	Sensitivity (%)	Specificity (%)
Dudley *et al.* [85]	2016	Prospective	USA	74	31	Brushing	39 gene panel	74.0	98.0
Singhi *et al.* [81]	2020	Prospective	USA	220	150	Brushing/Biopsy	28 gene panel	73.0	100.0
Harbhajanka *et al.* [86]	2020	Prospective	USA	94	43	Brushing	52 gene panel^a^	93.0	100.0
Arechederra *et al.* [87]	2022	Prospective	Spain	68	33	Bile	52 gene panel^a^	96.4	69.2
Kamp *et al.* [83]	2023	Retrospective	Netherlands	40	20	Brushing	14 gene panel	75.0	85.0
He *et al*. [88]	2023	Retrospective	China	105	64	Bile	23 gene panel	78.0	100.0
		Prospective		50	40	Bile	23 gene panel	75.0	90.0
Meijering *et al.* [89]	2025	Retrospective	Netherlands	94	63	Brushing	41 gene panel	56.0	100.0
				15	12	Biopsy	41 gene panel	58.0	100.0
Bardhi *et al.* [82]	2025	Retrospective	USA	77	28	Brushing/Bile	28 gene panel	75.0	95.9

a Oncomine Pan-Cancer Cell‑Free Assay (from Thermo Fisher Scientific).

### Methylated DNA markers

Aberrant DNA methylation is an epigenetic modification that often leads to silencing of tumor suppressor genes and is highly specific for malignant transformation, making MDMs a robust biomarker for cancer detection [90]. Several studies have consistently shown that biliary brush–based MDMs markedly outperform cytology in sensitivity while maintaining high specificity. Various genes that have been identified to be differentially methylated in malignant biliary strictures have subsequently been incorporated in diagnostic panels (Table 5). In an early study of CCA, methylated *EMX1* and *HOXA1* showed 100% sensitivity and 92% specificity compared with non-PSC controls [92]. A four-gene methylation panel (*CDO1*, *CNRIP1*, *SEPT9*, *VIM*) detected CCA in a study of 103 biliary brushes from 92 patients achieving a sensitivity of 85% and specificity of 98% including in patients with PSC where performance of brush cytology was suboptimal [91]. A subsequent study has reported high performance for individual markers (e.g. *HOXA1*, *NEUROG1*) and strong accuracy for parsimonious multi-marker combinations in suspected biliary strictures [93]. In a larger prospective cohort, a four-marker panel (*HOXA1*, *TWIST1*, *VSTM2B*, *CLEC11A*) achieved 73.4% sensitivity and 92.9% specificity, substantially exceeding cytology sensitivity (20.6%) and showing performance comparable to FISH; importantly, combining MDMs with cytology further increased yield and surpassed the combination of cytology plus FISH in that study [80].

**Table 5:** Diagnostic performance of individual methylated DNA markers for malignant biliary strictures (2015 to present)

Study	Year	Study design	Country	Total cohort (*n*)	Individuals with malignancy (*n*)	Sample type	MDM tested	Sensitivity (%)	Specificity (%)
Andresen *et al.* [91]	2015	Retrospective	Norway, UK	103	49	Brushing	*CDO1*	77.0	N/A
							*CNRIP1*	70.0	N/A
							*SEPT9*	57.0	N/A
							*VIM*	45.0	N/A
							**MDM panel** ^a^	**85.0**	**98.0**
Yang *et al.* [92]	2021	Retrospective	USA	34	16	Brushing	*HOXA2*	100	92.0
							*EMX1*	100	92.0
Prachayakul *et al.* [93]	2022	Prospective	Thailand	67	41	Brushing	*HOXA1*	95.1	65.4
							*NEUROG1*	90.2	88.5
Cooley *et al.* [80]	2024	Prospective	USA	197	64	Brushing	*TWIST1*	59.4	95.3
							*HOXA1*	53.1	95.3
							*VSTM2B*	62.5	95.3
							*CLEC11A*	50	95.2
							**MDM panel** ^a^	**73.4**	**92.0**

MDM = methylated DNA marker, N/A = not applicable or not reported in the original article.

a MDM panel is positive when ≥1 MDM is positive.

Beyond diagnostic accuracy, MDM assays offer practical advantages over FISH, including lower cost, reduced dependence on specialized expertise, and elimination of subjective interpretation or interobserver variability [94]. These features make MDM well suited for broad implementation across centers. They represent a scalable and reliable adjunct to cytology and biopsy to improve the diagnostic performance of these traditional modalities [1, 33].

### Bile as a liquid biopsy specimen

Bile has been explored as a liquid biopsy matrix for diagnosis of pancreatobiliary malignancies as it directly interfaces with the biliary epithelium. Cell-free DNA concentration in bile may exceed that in plasma, increasing the likelihood of detecting tumor-derived material [87].

Even before molecular testing, bile cytology can complement brushing: in a cohort of 239 patients, brush cytology alone (62.5%) and bile aspirate cytology alone (56.4%) had limited sensitivity, but the combination increased sensitivity to 81% while maintaining 100% specificity [95]. The diagnostic yield may be further improved by sampling bile after endoscopic interventions, leveraging epithelial disruption to increase cellular recovery; one study reported higher sensitivity for post-brushing bile cytology than for bile aspirate cytology (70% vs 34%) with 100% specificity [96].

Bile also supports multiple molecular layers of testing. Bile-derived NGS panels have reported sensitivities of 73%–96% and specificities of 69%–100% for malignancy detection, with sustained performance in PSC (reported sensitivity 75%–83%) [87]. Similarly, bile-based methylation assays have evolved from early promoter methylation targets with moderate sensitivity to multi-gene panels with substantially higher sensitivity for extrahepatic CCA [97]. More recently, in a pilot study, a four-gene methylation panel (*CDO1*, *CNRIP1*, *SEPT9*, *VIM*) accurately detected CCA in PSC up to 12 months before clinical diagnosis [98]. Proteomic profiling of bile has also shown promise for distinguishing malignant from benign strictures. Specific protein panels identified in bile have shown strong discriminatory power for CCA and pancreatic adenocarcinoma, suggesting potential utility for early and noninvasive diagnosis, though these findings remain investigational and require large-scale validation [99].

Bile aspiration can easily be performed during routine ERCP and thus can be incorporated into existing practice. Bile-based diagnostics can serve as adjunctive platform to pair with standard tissue sampling, either to increase sensitivity when brush/biopsy is nondiagnostic, to enable molecular detection when cellularity is limited, or to support longitudinal surveillance strategies in high-risk populations such as PSC. Bile, however, as a specimen cannot be used for targeting precise biliary location or mapping of malignancy due to its contact with the entire biliary tree.

## Conclusions

Timely and accurate diagnosis of malignant biliary strictures remains a challenge due to limited sensitivity of current diagnostic tools. Accordingly, contemporary practice has shifted from single-test strategies to layered, context-specific sampling pathways that prioritize timely diagnosis while minimizing repeat procedures and delays in definitive management. At index ERCP, brush cytology should be paired with either intraductal biopsy or with additional adjunctive testing (e.g. FISH) when available. This multi-modal approach of combining at least two modalities is strongly recommended by multiple guidelines [1, 31–33]. When standard sampling remains inconclusive, POC provide the most direct route to targeted tissue acquisition and lesion characterization. Developments in AI-assisted cholangioscopy have shown promising results to eliminate interobserver variability and allow for precise tissue sampling. Molecular diagnostic techniques applied to ERCP specimens (e.g. NGS and MDMs) are increasingly utilized to raise diagnostic confidence, and allow for precision diagnostics [100]. Adjunct technologies, including bile-based liquid biopsy, represent exciting future directions with potential for early malignancy detection, though further large, multicenter validation studies are necessary. In conclusion, integrating advanced molecular and imaging tools during ERCP when available can enhance diagnostic accuracy of malignant biliary strictures and guide early management.

## Authors’ contributions

All authors contributed to the conception and design of this review. J.K. and C.G. conducted the literature search and prepared the initial draft. M.A. critically revised the manuscript for important intellectual content. V.C. critically revised the manuscript and provided supervision. All authors reviewed and approved the final manuscript.
